# Evaluation Effects of Laser Therapy and Extracorporeal Shock Wave Therapy with Clinical Parameters and Magnetic Resonance Imaging for Treatment of Plantar Fasciitis in Patients with Spondyloarthritis: A Randomized Controlled Trial

**DOI:** 10.1155/2020/4386361

**Published:** 2020-08-27

**Authors:** Kezban Armagan Alpturker, Ayse Beyhan Lale Cerrahoglu, Ihsan Sebnem Orguc

**Affiliations:** ^1^Department of Physical Medicine and Rehabilitation, Division of Rheumatology, Celal Bayar University Medical School, Manisa, Turkey; ^2^Department of Physical Medicine and Rehabilitation, Celal Bayar University Medical School, Manisa, Turkey; ^3^Department of Radiodiagnostics, Celal Bayar University Medical School, Manisa, Turkey

## Abstract

**Objective:**

Low-level laser therapy (LLLT) and extracorporeal shock wave therapy (ESWT) is applied in the conservative treatment of inflammatory plantar fasciitis, which is also a characteristic feature of spondyloarthritis (SpA) (Gill, 1997 and Roxas, 2005). We determined and compared the effectiveness of LLLT and ESWT using magnetic resonance imaging (MRI).

**Methods:**

This study is a prospective, randomized, comparative, single-blind clinical study. Voluntarily followed 40 patients with the diagnosis of SpA and having pain at the heels at least for 6 months. Patients were divided randomly into two treatment groups. One group undertook 14 sessions of infrared Ga-Al-As LLLT, and the other group undertook 3 sessions ESWT. Feet functions of the patients were evaluated by American Orthopaedic Foot and Ankle Society (AOFAS) and Roles and Maudsley Scoring; VAS was evaluated for foot pain and function. In clinical assessment, disease activity was carried out by applying the BASDAI, the functional assessment was evaluated through the BASFI, and the patient quality of life was evaluated through the ASQoL; enthesitis was scored according to MASES assessment, performed before and at 1 month after treatment. The thickness of the plantar fascia was measured with MRI before and 1 month after treatment.

**Results:**

Compared with the pretherapy, progress in the feet function by AOFAS and Roles-Maudsley scoring and decrease in VAS levels were statistically significant in both groups (*p* < 0.001). Only the VAS exercise score was superior to LLLT (*p* < 0.05). The thickness of the plantar fascia had decreased significantly on MRI in all two groups.

**Conclusion:**

The treatment of plantar fasciitis with LLLT and ESWT was more successful in pain improvement and functional outcomes with the dose, frequency, and duration used in our study.

## 1. Introduction

Plantar fasciitis (PF), a frequent cause of heel pain, is a chronic inflammatory disorder of the fascial enthesis. Mechanic, degenerative, inflammatory, and traumatic causes are defined as enthesopathy while inflammatory enthesopathy is called “enthesitis” [1–3]. The clinical course of axial spondyloarthritis (SpA) is variable and characterized by spinal involvement and extraspinal manifestations, such as peripheral enthesitis [3, 4]. Peripheral enthesitis is most commonly found in the calcaneus plantar fascia and the Achilles tendon. It is thought that the repetitive mechanical loads and tendon movements are the reason why it is most often seen in the lower extremity. In addition to the biomechanical factor, the presence of HLA-B27 plays an important role in the development of enthesitis [5, 6]. Clinical diagnosis of plantar fasciitis is based on anamnesis and physical examination. In the early period of the disease, it can be detected normally with an X-ray. Magnetic resonance imaging (MRI), although expensive, is a very sensitive imaging method to evaluate plantar fascia morphology and bone marrow edema [7]. Major MRI findings include increased thickness of plantar fascia (>4 mm) pattern at T1-weighted images, diffuse bone marrow edema at the calcaneal adhesion site, and soft tissue edema around the pattern at T2-weighted images.

Typical pain, especially with the first few steps in the morning or after prolonged, present with a throbbing, burning, or piercing type of inferior heel pain. The treatment of plantar fasciitis is mainly conservative. Conservative treatment options include weight loss, Achilles tendon and fascia stretching exercises, daily physical activity arrangements, night supports, hot-cold treatments, ice massage, and soft and orthopedic walking shoes; viscoelastic ground still and physical therapy methods are used in chronic-resistant cases (therapeutic ultrasound therapy, iontophoresis, shock wave therapy, and low-level laser therapy) [2, 8, 9].

ESWT is the combination of the first letters of the words of Extracorporeal Shockwave Therapy [10]. The possible mechanism of ESWT in soft tissues is thought to be the release of growth factors associated with angiogenesis after shockwave application and accelerate tissue healing by increasing the formation of new vessels and oxygenation in the environment [11, 12].

The basic mechanism of low-level laser therapy (LLLT) is tissue stimulation. Biostimulant means accelerating the self-repair activity of living tissue. This effect is caused by the lymphatic drainage effect of radiation applied to tissues. The LLLT increases the permeability of the cell membrane and accelerates the fibroblast activity by increasing cell metabolism [13].

Despite the increasing popularity of LLLT and ESWT, randomized controlled studies comparing the efficacy of treatment modalities are lacking. This study is aimed at comparing and determining the anti-inflammatory analgesic effect of LLLT and ESWT at a 1-month follow-up of plantar fasciitis in patients with spondyloarthritis.

## 2. Patients and Methods

**Figure 1:**
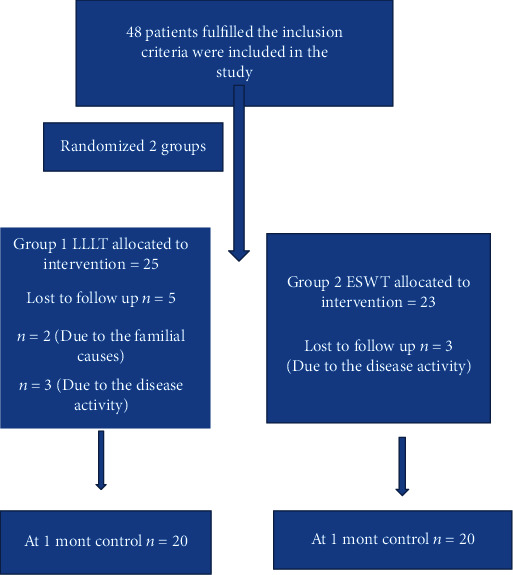
Flowchart of the study design. Abbreviations: ESWT: extracorporeal shock wave therapy; LLLT: low-level laser therapy.

The study group included 48 people who responded to the invitation to participate and agreed to be involved in the proposed research study. At the end of the treatment, they were told to come to check after a month. However, 40 people completed the study (20 males, 20 females; mean age 37.78 ± 9.86 years; range 18 to 60 years). Study protocol of Celal Bayar University Faculty of Medicine Ethics Committee was taken with the decision No: 20478486; after the decision, patients were included in the study. Written informed consent was obtained from each patient. The study was conducted under the principles of the Declaration of Helsinki. Statistical power analysis was carried out before starting the study. The minimum number of people (*n*) for each group was calculated to be 20 when divided into 2 groups with 95% confidence, 80% effect size, and 70% power (Figure 1).

The involvement criteria were as follows: the diagnosis of SpA by the criteria of Assessment of Spondyloarthritis International Society (ASAS) 2009, without the active disease (BASDAI < 4, without ESR and CRP elevation in routine control), having at least 6 months of heel pain. The diagnosis was confirmed clinically by the physical examination finding of tenderness to palpation with local pressure at the origin of the plantar fascia on the medial tubercle of the calcaneus and with passive dorsiflexion of fingers. In our study, patients' pain (after waking up in the morning in the first few step or increased pain during walking after resting) was evaluated with a visual analog scale (VAS). An indication of significant pain by a score of >5 for VAS (VAS for the first few steps in the morning and during exercise).

The exclusion criteria were history of heel surgery, application of physical therapy methods, or local steroid injection in the heel within the last 3 months and any disease contraindicated to physical therapy. This was a prospective, laminated blocked randomized, comparative, single-blind, clinical study. The first treatment group was determined by the closed-envelope randomization method of the first patient meeting the criteria. Then two-block randomization was performed by minimization method stratification according to age and sex.

14 sessions of 50 mW, 10 Hz, 8 J/cm^2^, and 830 nm wavelength infrared Ga-Al-As LLLT was applied to one group; 3 sessions in total of 10 Hz frequency 2.5 bar, and 2000 beat shock wave was applied once a week to the other group. LLLT, US therapy, and ESWT were performed by the same investigator using the BTL-5000 SWT combined device (BTL Turkey, Ankara, Turkey). All patients were included in the supervised exercise program.

American Orthopaedic Foot and Ankle Society (AOFAS) is a rearfoot score questionnaire that comprises nine questions to evaluate pain, function, and foot alignment. It has evaluated over a total of 100 points. A result of 0-69 bad, 70-79 moderate, 80-89 good, and 90-100 is considered excellent [14].

Roles-Maudsley scoring system is a practical scoring method that measures the association of pain on the extremity with activity. The pain is scored as excellent-good-medium and bad [14].

Pain intensity during exercise and morning first step was measured on a 100 mm visual analog scale (VAS). The endpoints of the scale were determined by no pain at 0 mm and unbearable pain at 100 mm [15].

ASQoL is a scale that questions the quality of life of patients by giving yes-no answers to each question out of 18 questions. The sum of yes gives the score. Lower scores indicate better quality of life [16].

BASDAI is an index of disease activity. There are 6 questions about fatigue, spinal and peripheral joint pain, compression sensitivity, and morning stiffness. On the 10 cm horizontal VAS scale, a value of 0 to 10 is determined in the first five questions, and the average of these two questions related to morning stiffness is averaged and the first 4 questions are summed and divided by 5 to produce 0-10 points. A score of 4 or more indicates active disease [17].

Bath Ankylosing Spondylitis Functional Index (BASFI) consists of 10 questions, and each question is given a score of 0-10. Eight of them are related to activities of daily living and two of them evaluate coping with daily life. BASFI's total score is calculated by averaging 10 questions [17].

MRI was performed using a SIGNA_ HDXT 1.5 Tesla MRI system (GE Healthcare, Chicago, IL) before and 1 month after treatment. The maximum thickness of the proximal plantar fascia where it attaches to the calcaneus was measured using electronic calipers on fluid-sensitive MRI sequences. After all the patients had completed the therapy, the pre- and posttreatment MRI scans were interpreted simultaneously by a radiologist (S.O.), who was unaware of the treatment groups.

### 2.1. Statistical Analysis

All analyses were performed using SPSS for Windows version 15.0 software (SPSS Inc., Chicago, IL, USA). Continuous variables with normal distribution were shown as mean ± standard deviation. Categorical variables were shown as numbers. To compare the two groups for continuous variables, the groups were compared using the *t*-test for independent samples and the Mann–Whitney *U* test for categorical data. A *p* value of less than 0.05 was considered statistically significant.

## 3. Results

Demographical data and measurement results of the involved patients were presented in Table 1.

The mean of Roles-Maudsley scoring, AOFAS posterior foot score, and VAS values calculated before and after treatment of the patients in the LLLT group is given in Table 2. Results were statistically significant in favor of treatment (*p* < 0.05).

The mean of Roles-Maudsley scoring, AOFAS posterior foot score, and VAS values calculated before and after treatment of the patients in the ESWT group is given in Table 3. Results were statistically significant in favor of treatment (*p* < 0.05).

In Table 4, when the differences between pre and posttreatment were examined, the difference in favor of treatment was higher in the ESWT group, but there was no statistical superiority to the LLLT (*p* > 0.05). In both groups, AOFAS posterior foot score was bad but increased to moderate after treatment.

There was no significant difference between the two groups in mean VAS score first steps in the morning (*p* > 0.05). The decrease in the mean VAS value in the ESWT group during exercise was more in favor of treatment, and the difference was statistically significant (*p* < 0.05). Only the VAS exercise difference was superior to the LLLT group (*p* < 0.05).

In Table 5, the mean of the evaluations are performed to investigate the effect of SpA cases on life activities and functions of the patients and to determine the disease activity are given in the table. When the intragroup evaluation was performed in the LLLT group, a statistically significant difference was found in all parameters compared to before the treatment (*p* < 0.05).

In Table 6, the intragroup evaluation was performed in the ESWT group, a statistically significant difference was found in all parameters compared to before treatment (*p* < 0.05).

In Table 7, when the difference between the mean values was evaluated between the groups, the difference was found to be greater in the ESWT group compared to the LLLT group, but these differences were not statistically significant (*p* > 0.05).

In Table 8, the mean thickness of the plantar fascia measured by MRI in the laser group was 4.43 ± 0.984 (min: 3.6 mm, max 8.50 mm) before treatment and 3.66 ± 0.613 (min: 2.9 mm, max: 5.50 mm) after treatment. In the ESWT group, the mean thickness of the plantar fascia measured by MRI was 4.50 ± 0.421 (min: 3.80 mm, max: 5.40 mm) before treatment and 3.75 ± 0.423 (min: 3.0 mm, max: 4.50 mm before treatment), respectively. This decrease was found to be statistically significant after treatment (*p* > 0.05). The difference of plantar fascia thickness was slightly higher in the LLLT group compared to the ESWT group, but this difference was not statistically significant (*p* > 0.05).

In Table 9, the correlation between MASES and BASFI and BASDAI was found to be statistically significant and moderately positive (*p* < 0.05). There was a statistically significant positive correlation between SpA disease duration and BASMI (*p* < 0.05). The correlation between plantar fascia thickness and BASFI and BASDAI was found to be moderately positive (*p* < 0.05). The correlation between the duration of plantar fasciitis and the pretreatment values of BASDAI was statistically significant and moderately positive (*p* < 0.05).

## 4. Discussion

Plantar fasciitis is a chronic inflammatory disorder of the fascial enthesis. Enthesitis is a distinctive pathological feature of spondyloarthritis [18]. The most important symptom of plantar fasciitis is pain. Physical therapy agents play an important role in relieving pain and inflammation in SpA.

In this randomized study, patients followed with SpA diagnosis and having heel pain at least 6 months, diagnosed with PF differentially by physical examination, and with symptoms that did not regress with first step conservative treatment are divided into two groups randomized.

The mean of the BMI from the demographical data of the patients was 27.60 ± 5.05 kg/m^2^; the patients were overweighted according to World Health Organization classification (BMI: 25.00-29.99, overweighted). The most important mechanical factor leading to plantar fasciitis is overweight [19]. In a study search for the relationship between obesity and SpA, overweighted obese SpA patients are stated to be with less success in treatment than normal-weight SpA patients [20].

The incidence of plantar fasciitis increases with age. Rudwaleit et al. performed a study with patients of AS and axial SpA. The mean age of patients with axial SpA was 36.1 ± 10.6 years, and the average age of onset of disease was 33.2 ± 10.5 [21]. In our study, the mean age of the patients was 37.78 ± 9.86 years, and the average age of onset of disease was 31.45 ± 9.86 years. So we found that demographic values are close to those in the literature.

There are many physical therapy methods used in conservative treatment of plantar fasciitis but in the last years especially LLLT and ESWT have gained popularity in recent years [2].

In the 2014 heel pain guide, LLLT indicated reduced heel pain and limitation of foot activity, and it was recommended for plantar fasciitis treatment at the level of C evidence [22]. One of the Cochrane studies associated with the pain-reducing effect of LLLT therapy (LLLT) in the rheumatologic diseases states that it was an effective way to decrease the pain and morning stiffness [23].

In animal studies, different doses of LLLT were examined and 50 mW was found to be more effective on inflammatory mediators such as IL-1*β* and IL-6 and inflammatory cell inhibition than 100 mW [24]. IL-1 and TNF-*α* play an important role in inflammation in SpA, a chronic inflammatory disease. Our study is aimed at showing that LLLT has an analgesic effect on neurotransmitter secretion such as serotonin, and additionally, it has an anti-inflammatory effect by reducing TNF-*α* levels. A similar dose (50 mW) has been applied in the studies and the correlation of the disease activity parameters with clinical improvement is consistent with the data in our study [25].

Harjacek et al. applied LLLT Ga-Al-As with 2.5-3 J/m^3^ dose to enthesitis regions of 20 juvenile SpA patients with plantar fasciitis (12 girls, 8 boys, mean age 11.4) and one month after treatment the pain level was evaluated by VAS. VAS level was significantly decreased (decreased from 6 to 1.3). As a result of this study, the anti-inflammatory, analgesic, and antiedematous effectiveness of LLLT were emphasized [26]. This study was similar to our study in terms of using VAS and was performed in inflammatory plantar fasciitis.

A large number of randomized controlled trials were done to investigate ESWT efficacy in the treatment of plantar fasciitis. Shock waves have local anti-inflammatory effects. It has been shown that shock waves used in wound healing increase new vessel formation, regeneration, and create an antimicrobial environment [27].

In a meta-analysis published in 2013, it was stated that ESWT had an effect on pain and function in tendinopathies and no side effects were seen. However, there is no complete and standard consensus on the implementation protocol of ESWT. In the treatment of chronic plantar fasciitis, ESWT decreased pain scores and its effect was reported to last for 12 months. ESWT is reported to be useful as a safer and more effective method in chronic cases [28]. The patients in our study were also chronic cases whose symptoms persisted despite other conservative treatments for at least 6 months and similar to the literature; we found that the treatments were effective. In our study, the effect of ESWT on pain is evaluated by VAS and the pain score of the individuals during exercise was significantly decreased (*p* = 0.001). We found ESWT superior to LLLT when we compare the two groups (*p* = 0.011).

Cosentino et al. investigated the efficacy of ESWT for calcaneal enthesopathy in 60 patients with heel pain and observed a significant decrease in VAS. Pain scores at rest and during activity 1 and 3 months after treatment. Changes in the entophyte structure were evaluated by ultrasonography, and no statistically significant difference was observed immediately after the treatment, but after 1 month there was a significant difference. He stated that ESWT may be effective in reducing pain, inflammation, edema, and altering entophyte morphology with this study [29].

In a study of patients with chronic plantar fasciitis, endoscopic surgery was compared with ESWT (0.22 mJ/mm^2^, total 1,500 shocks, 4 sessions maximum painful point, and around 2 cm area) and evaluated with American Orthopaedic Foot and Ankle Society (AOFAS) and Roles-Maudsley scoring. It was found that it provided tissue healing with controlled inflammation and the effects started on the 3rd week after the treatment and the effects continued until one year. It has been reported that ESWT may be a noninvasive treatment that should be performed before surgery. In a study comparing ESWT and surgery, patients were called for a check-up after 3 weeks to assess treatment efficacy [30]. As we aimed to show the response to treatment and to show the efficacy of the treatment, the controls were performed at 1 month after the treatment.

Enthesopathy has been reported to be the most important factor affecting the quality of life in the inflammatory disease [31]. In our study, VAS, ASQoL, BASDAI, BASFI, and MASES values used to evaluate pain, functionality, and disease activity of SpA patients were significantly decreased after treatment (*p* < 0.05). In a study investigating the effect of LLLT on pain and functionality in AS patients, individuals (*n* = 37) were divided into two groups; Ga-Al-As LLLT with a power of 30 mW, *n* = 18 and the other group (*n* = 18) underwent placebo LLLT application and VAS, BASDAI, BASFI, and ASQoL were used as scales. VAS (*p* < 0.05), ASQoL (*p* < 0.01), and BASDAI (*p* < 0.001) showed significant improvement at the end of treatment and at 2 months after treatment (*p* < 0.05). However, no statistically significant difference was found between treatment and placebo groups [32].

In the study, comparing some variables with BASDAI, it was found that the presence of enthesitis reflected active disease and was higher in SpAs with enthesitis [33]. We included patients with BASDAI < 4 in our study.

In the study of Zhu et al., MRI was performed to monitor acute (after 24 hours) changes after a single session of 1500 shock wave pulsed ESWT treatment on the patients with chronic plantar fasciitis. Plantar fascia thickness and spur were examined with T1 sequence; the presence of calcaneal bone marrow edema and soft tissue edema were examined with T2 fat-suppressed sequence. After ESWT administration, soft tissue edema increased and plantar fascia thickness did not change in the acute period [34]. This study is important for us in terms of showing the revascularization effect of ESWT in the acute phase and the use of MRI.

To investigate the relationship between SpA disease duration and patients' quality of life, disease activity, and metrological measurements, we looked at the correlation between ASQoL, BASDAI, and BASMI. We found that the correlation between SpA disease duration and BASMI was statistically significant and correlated positively with each other (*p* < 0.05). We thought that our patients were exposed to inflammation with prolonged duration of the disease and consequently increased joint movements.

In our study, the correlation of disease parameters was evaluated and the result was statistically significant. A moderate positive correlation was found between MASES, BASFI, and BASDAI. Similarly, in another study evaluating AS patients with enthesitis, there was a correlation between MASES and BASFI, BASDAI, and Short form-36 (SF-36), but any correlation was found between disease duration and laboratory values [35]. In our study, there was no significant correlation between MASES and SpA disease duration and plantar fasciitis duration. As mentioned previously, sometimes enthesitis pain can be the first symptom of SpA.

We found anti-inflammatory and analgesic effects of LLLT and ESWT statistically significant as it was the primary aim of our study. For the second aim of the study, we found significant results in both of the groups when we correlated the parameters of disease evaluation with clinical parameters. We did not observe any side effects of the treatment methods.

## 5. Conclusion

We used clinical scales to evaluate the efficacy of LLLT and ESWT in the treatment of inflammatory plantar fasciitis. We found significant results in favor of treatment in both groups. These findings suggest that LLLT and ESWT may be beneficial in the treatment of plantar fasciitis in the dose, frequency, and duration we used in our study.

In conclusion, we found that LLLT and ESWT significantly reduced the pain experienced with plantar fasciitis, providing clinical improvement.

## 6. Study Limitations

The present study had several limitations. The first and most important limitation was the short follow-up period. Second, the sample size of the study was relatively limited. We could not include a placebo group because of ethical concerns.

With this study, we wanted to state that plantar fasciitis, which is common in patients with Spondilaoarthritis, can be treated with electrotherapy, and its effectiveness can be demonstrated by clinical and imaging methods.

It is a study that may be important in terms of demonstrating the effectiveness of physical therapy methods with clinical and imaging methods.

## Figures and Tables

**Table 1:** Characteristics of the patients in the two study groups.

		LLLT group	ESWT group	*p* value	All patients
		*n* (%)	*n* (%)		*n* (%)
Sex	Men	10 (50%)	10 (50%)	1.00	20(50%)
	Women	10 (50%)	10 (50%)		20(50%)
Age		37.00 ± 10.33	38.55 ± 9.57	0.620	37.78 ± 9.86
BMI (min–max)		27.15 ± 4.99 (20.42-35.94)	28.36 ± 5.17 (20.31-38.29)	0.469	27.60 ± 5.05 (20.31-38.29)
SpA period (year) (min–max)		5.68 ± 5.11 (1.5-18)	5.35 ± 4.87 (2-20)	0.838	5.51 ± 4.93 (1.5-20)
AS		4 (20%)	3 (15%)		7 (17.5%)
PsA		3 (15%)	3 (15%)		6 (15%)
EA		0	2 (10%)	*p* > 0.05	2 (5%)
ReA		2 (10%)	0		2 (5%)
uSpA		11 (55%)	12 (60%)		23 (57.5%)
Heelpainduration		18.75 ± 14.96	18.90 ± 16.68	0.976	18.83 ± 15.64
(month)					
(min–max)		(6-48)	(6- 60)		(6-60)

Abbreviations: BMI: body mass index; AS: ankylosing spondylitis; PsA: psöriatic arthritis: EA: enteropathic arthritis; ReA: reactive arthritis; uSpA: undifferentiated spondyloarthritis.

**Table 2:** Pre- and posttreatment AOFAS posterior foot score, RMS score, and VAS score first steps in the morning and VAS exercise results in the LLLT group (*n*: 20).

	Pretreatment	Posttreatment	*p* value
	Mean ± SD	Mean ± SD	
AOFAS posterior foot score	64.30 ± 8.856	75.25 ± 2.447	0.001
Roles-Maudley score	2.95 ± 0.51	2.00 ± 0.56	0.001
VAS score first steps in the morning	69.75 ± 8.025	38.50 ± 12.042	0.001
VAS exercise	75.50 ± 9.720	48.50 ± 12.258	0.001

**Table 3:** Pre- and posttreatment AOFAS posterior foot score, RMS score, and VAS score first steps in the morning and VAS exercise results in the ESWT group (*n*: 20).

	Pretreatment	Posttreatment	*p* value
	Mean ± SD	Mean ± SD	
AOFAS posterior foot score	63.90 ± 11.59	75.80 ± 8.78	0.001
Roles- Maudley score	3.00 ± 0.64	1.85 ± 0.58	0.001
VAS score first steps in the morning	67.75 ± 8.95	34.50 ± 9.58	0.001
VAS exercise	78.00 ± 7.67	44.00 ± 13.43	0.001

**Table 4:** Comparisons of the differences between posterior foot AOFAS before and after treatment, VAS score first steps in the morning and exercise, and Roles-Maudley score.

	LLLT group	ESWT group	*p* value
	Mean ± SD	Mean ± SD	
AOFAS difference	10.65 ± 6.19	11.90 ± 6.83	0.548
VAS exercise difference	26.75 ± 7.65	34.00 ± 8.36	0.011
VAS score first steps in the morning difference	31.00 ± 7.71	33.25 ± 4.94	0.279
Roles-Maudley score difference	0.95 ± 0.60	1.15 ± 0.48	0.257

**Table 5:** Results of clinical parameters evaluation in the LLLT group (*n*: 20).

	Pre-treatment	Posttreatment	*p* value
	Mean ± SD	Mean ± SD	
ASQoL	12.00 ± 2.865	8.80 ± 2.375	0.001
BASFI	3.63 ± 0.75	3.05 ± 0.585	0.001
BASDAI	3.85 ± 0.480	3.57 ± 0.561	0.001
MASES	4.00 ± 1.55	3.20 ± 1.36	0.003

**Table 6:** Results of clinical parameters evaluation in ESWT group (*n*: 20).

	Pretreatment	Posttreatment	*p* value
	Mean ± SD	Mean ± SD	
ASQoL	11.45 ± 2.21	8.20 ± 2.66	0.001
BASFI	3.58 ± 0.850	2.94 ± 0.755	0.001
BASDAI	3.70 ± 0.512	3.31 ± 0.620	0.001
MASES	3.95 ± 1.14	3.55 ± 0.82	0.011

**Table 7:** Comparison of differences between groups of pre- and posttreatment results of ASQoL, BASDAI, BASFI, and MASES.

	LLLT group	ESWT group	*p* value
	Mean ± SD	Mean ± SD	
ASQoL difference	3.10 ± 1.61	3.25 ± 1.71	0.777
BASDAI difference	0.28 ± 0.23	0.38 ± 0.28	0.226
BASFI difference	0.57 ± 0.48	0.63 ± 0.37	0.663
MASES difference	0.80 ± 0.89	0.40 ± 0.59	0.104

**Table 8:** Magnetic resonance imaging findings in the LLLT and ESWT groups.

	Before treatment	After treatment	*p* value
	Mean ± SD	Mean ± SD	
Plantar fascia thickness (mm) in the LLLT group	4.43 ± 0.984	3.66 ± 0.613	0.001
Plantar fascia thickness (mm) in the ESWT group	4.50 ± 0.421	3.75 ± 0.423	0.001
Plantar fascia difference in the treatment groups (mm)	0.772 ± 0.549	0.750 ± 0.241	0.277

**Table 9:** MASES and SpA disease duration correlation between plantar fascia thickness in MRI, ASQoL, BASFI, and BASDAI.

	Plantar fascia thickness in MRI (mm)	MASES before treatment	SpA disease duration	Plantar fasciitis duration
ASQoL	*p*: 0.809	*p*: 0.656	*p*: 0.140	*p*: 0.098
	*r*: -0.040	*r*: 0.073	*r*: 0.238	*r*: 0.256
BASFI	*p*: 0.035	*p*: 0.048	*p*: 0.113	*p*: 0.089
	*r*: 0.335	*r*: 0.314	*r*: 0.255	*r*: 0.272
BASDAI	*p*: 0.035	*p*: 0.008	*p*: 0.385	*p*: 0.012
	*r*: 0.335	*r*: 0.414	*r*: 0.141	*r*: 0.392

*r*: correlation coefficient (0-0.25 = weak, 0.25-0.50 = medium, 0.50-0.75 = strong, and 0.75-1 = very strong).

## Data Availability

All the authors allow the data are fully available without restriction.

## References

[1] Gill L. H. (1997). Plantar fasciitis: diagnosis and conservative management. *The Journal of the American Academy of Orthopaedic Surgeons*.

[2] Roxas M. (2005). Plantar fasciitis: diagnosis and therapeutic considerations alternative medicine. *Review*.

[3] Kumai T., Benjamin M. (2002). Heel spur formation and the subcalcaneal enthesis of the plantar fascia. *The Journal of Rheumatology*.

[4] Ozgocmen S., Khan M. A. (2012). Current concept of spondyloarthritis: special emphasis on early referral and diagnosis. *Current Rheumatology Reports*.

[5] Poggenborg R. P., Eshed I., Østergaard M. (2015). Enthesitis in patients with psoriatic arthritis, axial spondyloarthritis and healthy subjects assessed by 'head-to-toe' whole-body MRI and clinical examination. *Annals of the Rheumatic Diseases*.

[6] Eshed I., Bollow M., McGonagle D. G. (2007). MRI of enthesitis of the appendicular skeleton in spondyloarthritis. *Annals of the Rheumatic Diseases*.

[7] Abreu M., Chung C., Mendes L., Mohana-Borges A., Trudell D., Resnick D. (2003). Plantar calcaneal enthesophytes: new observations regarding sites of origin based on radiographic, MR imaging, anatomic, and paleopathologic analysis. *Skeletal Radiology*.

[8] Ordahan B., Karahan A. Y., Kaydok E. (2018). The effect of high-intensity versus low-level laser therapy in the management of plantar fasciitis: a randomized clinical trial. *Lasers in Medical Science*.

[9] Skopljak A., Muftic M., Sukalo A., Masic I., Zunic L. (2014). Pedobarography in diagnosis and clinical application. *Acta Informatica Medica*.

[10] Ogden J. A., Toth-Kischkat A., Schultheiss R. (2001). Principles of shock wave therapy. *Clinical Orthopaedics and Related Research*.

[11] Rompe J. D., Kirkpatrick C. J., Kullmer K., Schwitalle M., Krischek O. (1998). Dose-related effects of shock waves on rabbit tendo Achillis. *Journal of Bone and Joint Surgery British Volume*.

[12] Türkoğlu G., Karahan A. Y., Akkurt H. E. (2017). Extracorporeal shockwave therapy versus kinesiology taping in the management of plantar fasciitis: a randomized clinical trial. *Archives of Rheumatology*.

[13] Kalyon T. A., Tuna N. (1989). LLLT. Ġç. *Elektroterapi*.

[14] Akbaba Y. A., Celik D., Ogut R. T. (2016). Translation, cross-cultural adaptation, reliability, and validity of Turkish version of the American Orthopaedic Foot and Ankle Society Ankle-Hindfoot Scale. *The Journal of Foot and Ankle Surgery*.

[15] Price D. D., McGrath P. A., Rafii A., Buckingham B. (1983). The validation of visual analogue scales as ratio scale measures for chronic and experimental pain. *Pain*.

[16] Duruöz M., Doward L., Turan Y. (2013). Translation and validation of the Turkish version of the ankylosing spondylitis quality of life (ASQoL) questionnaire. *Rheumatology International*.

[17] Zochling J. (2011). Measures of symptoms and disease status in ankylosing spondylitis: ankylosing Spondylitis Disease Activity Score (ASDAS), Ankylosing Spondylitis Quality of Life Scale (ASQoL), Bath Ankylosing Spondylitis Disease Activity Index (BASDAI), Bath Ankylosing Spondylitis Functional Index (BASFI), Bath Ankylosing Spondylitis Global Score (BASG), Bath Ankylosing Spondylitis Metrology Index (BASMI), Dougados Functional Index (DFI), and Health Assessment Questionnaire for the Spondylarthropathies (HAQ-S).

[18] Jacobs J. C. (1983). Spondyloarthritis and enthesopathy. Current concepts in rheumatology. *Archives of Internal Medicine*.

[19] Ulusoy A., Cerrahoglu L., Orguc S. (2017). Magnetic resonance imaging and clinical outcomes of laser therapy, ultrasound therapy, and extracorporeal shock wave therapy for treatment of plantar fasciitis: a randomized controlled trial. *The Journal of Foot and Ankle Surgery*.

[20] Gremese E., Bernardi S., Bonazza S. (2014). Body weight, gender and response to TNF-*α* blockers in axial spondyloarthritis. *Rheumatology*.

[21] Rudwaleit M., Haibel H., Baraliakos X. (2009). The early disease stage in axial spondylarthritis: results from the German spondyloarthritis inception cohort. *Arthritis & Rheumatism*.

[22] Martin R. L., Davenport T. E., Reischl S. F. (2014). Heel pain—plantar fasciitis: revision 2014. *Journal of Orthopaedic & Sports Physical Therapy*.

[23] Brosseau L., Welch V., Wells G. (2000). Low level laser therapy (classes I, II and III) in the treatment of rheumatoid arthritis. *Cochrane Database of Systematic Reviews*.

[24] Marcos R. L., Leal-Junior E. C., Arnold G. (2012). Low‐level laser therapy in collagenase‐induced Achilles tendinitis in rats: Analyses of biochemical and biomechanical aspects. *Journal of Orthopaedic Research*.

[25] Laatiris A., Amine B., Ibn Yacoub Y., Hajjaj-Hassouni N. (2012). Enthesitis and its relationships with disease parameters in Moroccan patients with ankylosing spondylitis. *Rheumatology International*.

[26] Harjacek M., Kelava T., Lamot L. (2008). The therapeutic value of low-energy LLLT (LLLT) for enthesitis in children with juvenile spondyloarthropathies. *Pediatric Rheumatology*.

[27] Mittermayr R., Antonic V., Hartinger J. (2012). Extracorporeal shock wave therapy (ESWT) for wound healing: technology, mechanisms, and clinical efficacy. *Wound Repair and Regeneration*.

[28] Aqil A., Siddiqui M. R., Solan M., Redfern D. J., Gulati V., Cobb J. P. (2013). Extracorporeal shock wave therapy is effective in treating chronic plantar fasciitis: a meta-analysis of RCTs. *Clinical Orthopaedics and Related Research*.

[29] Cosentino R., Falsetti P., Manca S. (2001). Efficacy of extracorporeal shock wave treatment in calcaneal enthesophytosis. *Annals of the Rheumatic Diseases*.

[30] Radwan Y. A., Mansour A. M. R., Badawy W. S. (2012). Resistant plantar fasciopathy: shock wave versus endoscopic plantar fascial release. *International Orthopaedics*.

[31] Turan Y., Duruöz M. T., Cerrahoğlu L. (2007). Quality of life in patients with ankylosing spondylitis: a pilot study. *Rheumatology International*.

[32] Aydin E., Gündüz O. H., Akcan E., Akyüz G. (2013). Effectiveness of low level LLLT therapy on pain and functional status in ankylosing spondylitis. *Turkish Journal of Physical Medicine & Rehabilitation*.

[33] Rezvani A., Bodur H., Ataman Ş. (2013). Correlations among enthesitis, clinical, radiographic and quality of life parameters in patients with ankylosing spondylitis. *Modern Rheumatology*.

[34] Zhu F., Johnson J. E., Hirose C. B., Bae K. T. (2005). Chronic plantar fasciitis: acute changes in the heel after extracorporeal high-energy shock wave therapy--observations at MR imaging. *Radiology*.

[35] Luo G., Zhao Z., Zhu J., Zhang J., Huang F. (2014). The clinical analysis for the whole-spine magnetic resonance imaging of axial spondyloarthritis. *Zhonghua Nei Ke Za Zhi*.

